# Re: unusual intravesical foreign body in a young female migrated from the vagina due to autoerotism

**DOI:** 10.1590/S1677-5538.IBJU.2017.0306

**Published:** 2017

**Authors:** Michael S. Floyd, Ahmad M. Omar, Altaf Q. Khattak

**Affiliations:** 1 Department of Reconstructive Urology, St Helens & Knowsley Hospital NHS Trust, Whiston Hospital, United Kingdom, UK

*To the editor,*

We read with interest the recent case of an unusual intravesical foreign body reported by Bansal et al. (1). A case is presented of an 18 year old female who presented with lower tract symptoms and was found to have a supratrigonal fistula following self insertion of a plastic pen per vagina for sexual gratification 6 months earlier. The operative management is described and high quality radiological and cystoscopic images are provided.

The authors allude to the array of intravesical bodies that have been reported and mention the psychological reasons for self insertion (1). It should be acknowledged that in certain patient cohorts, urethrovesical foreign body insertion is a form of manipulative behaviour as it requires mandatory transfer to an acute hospital (2) and that the practice is frequently mimicked by other institutionalised patients(3). Specific to the incarcerated population higher rates of emergency surgical intervention have been reported following urethral foreign body insertion (4).

The important role of radiology in determining the luceny, location and size of foreign bodies is discussed and the preference for endoscopic management is mentioned (1). The increasing role of the interventional radiologist in imaged guided retrieval of self inserted foreign bodies, should not be underestimated as illustrated by Young et al. (5).

The authors conclude by discussing urogenital fistulae as a consequence of foreign body insertion. Recent reports have highlighted the additional acute complication of urethral avulsion following polyembolokoilamania necessitating emergency urethroplasty (6).

Finally, it should be acknowledged that not all cases of self embedding behaviour require intervention as some patients deliberately request no intervention (7) and reports exist of cases that have been managed conservatively (8).
